# Indications and outcomes of emergency obstetric hysterectomy; a 5-year review at the Bafoussam Regional Hospital, Cameroon

**DOI:** 10.1186/s12884-021-03797-3

**Published:** 2021-04-23

**Authors:** Mbakwa Rickeins Mbakwa, Nicholas Tendongfor, Yannick Lechedem Ngunyi, Ekongefeyin Sintieh Nchinda Ngek, Frank Alemkia, Thomas Obinchemti Egbe

**Affiliations:** 1 Faculty of Health Sciences, University of Buea, Buea, Cameroon; 2 Microhealth Global Medical Center, Mbengwi, Cameroon; 3 Mbonge District Hospital, Mbonge, Cameroon; 4 Cameroon Baptist Convention Health Services, Yaounde, Cameroon; 5 Obstetrics and Gynaecology Service, Douala General Hospital, Douala, Cameroon

**Keywords:** Emergency obstetric hysterectomy, Caesarean hysterectomy, Postpartum hysterectomy, Complications, Indications, Predictors of adverse outcomes, Bafoussam Regional Hospital, Cameroon

## Abstract

**Background:**

Emergency Obstetric Hysterectomy (EOH) is removal of the uterus due to life threatening conditions within the puerperium. This life saving intervention is associated with life threatening complications. In our setting, little is known on EOH.

**Objectives:**

To determine the prevalence, indications and outcomes of emergency obstetric hysterectomy while comparing both postpartum hysterectomy and caesarean hysterectomy.

**Methods:**

A 5-year hospital-based retrospective cohort study involving medical records of patients who underwent emergency obstetric hysterectomies between 1st January 2015 and 31st December 2019, was carried out at the Bafoussam Regional Hospital (BRH) from 1st February 2020 to 30th April 2020. Cases were classified as caesarean hysterectomy (CH) or postpartum hysterectomy (PH). Epidemiological data, indications, and complications of EOH were collected and analyzed in EPI-INFO 7.2.2.1. The chi-squared test was used to compare the two groups, and bivariate analysis was used to identify indicators of adverse outcomes of EOH. Statistical significance was set at *p* < 0.05.

**Results:**

There were 30 cases of emergency obstetric hysterectomy (24 caesarean hysterectomies and 6 postpartum hysterectomies), giving a prevalence rate of 3.75 per 1000 deliveries. The most common indication for CH, was intractable postpartum haemorrhage and uterine rupture (33.33% each), while abnormal placentation (50%) was commonly indicated for PH. Anaemia (both groups) (*p =* 0.013) and sepsis (PH group only, 33.33%) (*p =* 0.03) were the most statistically significant complications of EOH respectively. Absence of blood transfusion prior to surgery (*p* = 0.013) and prolonged surgery lasting 2 or more hours (*p =* 0.04), were significantly associated with a negative clinical outcome.

**Conclusion:**

The prevalence of EOH is high. There were no differences in the sociodemographic profile, risk factors and indications of both groups. PH group was more likely to develop sepsis as complication. Lack of blood transfusion prior to surgery and prolonged surgeries were significantly associated to complication. Meticulous care and timely recognition of negative prognostic factors of delivery as well as those of EOH will help improve maternal outcomes of pregnancy.

## Background

EOH could be due to clinical indications such as life-threatening haemorrhage not responding to medical treatment, uterine rupture, abnormal placentation (placenta accreta) and sepsis. Emergency obstetric hysterectomy (EOH) is defined as the removal of the uterus either at the time of caesarean section (CS) or following vaginal delivery (VD) within the puerperium and it is usually performed in the face of life-threatening obstetric haemorrhage [1]. EOH is characterized with the dilemma of choosing between saving a life and sacrificing fertility, hence it is of importance this is prevented as much as possible. In Cameroon, despite the maternal mortality ratio (MMR) falling gradually from 749 deaths per 100,000 live births in 1998 to 467 deaths per 100,000 live births in 2018 [2] postpartum haemorrhage (PPH), a key indicator of EOH accounted for 29.2% of all maternal mortality [3]. Even though rendering the woman sterile, emergency obstetric hysterectomy is the bridge between life threatening postpartum haemorrhage and death. Emergency obstetric hysterectomy complicates almost 1 per 1000 deliveries world-wide ranging from 0.2–10.1 per 1000 births, with the prevalence higher in low and middle income, than upper middle and high income settings: 2.8 compared with 0.7 per 1000 deliveries respectively [4]. In Nigeria and Cameroon, the incidence of EOH are 5.1 and 1.25 per 1000 deliveries respectively with the incidence of Caesarean hysterectomies (CH) higher than postpartum hysterectomies (PH) in both settings [5, 6]. In Greece, placenta accreta accounted for 51.1% of EOH as compared to uterine rupture which accounted for 93.2 and 35% in Nigeria and Cameroon respectively [5–7]. Proper timing and meticulous care may reduce or prevent maternal complications. Despite the lifesaving intervention of the EOH, these patients must be monitored closely to prevent further complications such as wound infection, renal failure, disseminated intravascular coagulation (DIC), shock, septicemia and mortality [8]. Given that the incidence of this procedure is on the rise as a consequence to the rise in irreversible causes of life threatening haemorrhage, abnormal placentation and uterine rupture [9, 10], it requires utmost attention, as this will play a vital role to curb maternal mortality. With paucity of data on emergency obstetric hysterectomy in our setting, gradual drop in maternal mortality rate and the universal rising trend in Caesarean delivery as reported by WHO (12% in 2000 to 21% in 2015) [11], the importance of understanding the overall healthcare burden of EOH cannot be over-emphasized. Given our hypothesis that the prevalence of EOH is high, we therefore sort to assess the current trend of EOH in Cameroon and shed light on the prevalence, as well as comparing both postpartum hysterectomy and caesarean hysterectomy and its outcomes. This will help provide base line data, that could be useful for the formulation of better treatment guidelines, hence reducing maternal mortality in our setting.

## Methods

### Study design and setting

This was a hospital-based retrospective cohort study, conducted between 1st February 2020 to 30th April 2020 at the inpatient department of obstetrics and gynaecology of the Bafoussam regional hospital (BRH). The Bafoussam Regional Hospital (BRH), is located in the Bafoussam II subdivision, in the Mifi Division of the West Region of Cameroon. The BRH is a secondary healthcare facility that offers medical education, clinical care, and research to a population of over 1.5 million inhabitants of the city of Bafoussam and its environs. The Obstetrics and Gynaecology department has 3 obstetricians, 16 paramedical staff (comprising of midwives, nurses and assistant nurses), 3 outpatient consultation rooms, 1 delivery room, 5 delivery beds and 30 hospitalization beds with an annual average of 1500 deliveries.

### Study population and sampling

We included medical records of all pregnant women who gave birth at the BRH from 28 completed weeks of gestation to term and underwent hysterectomy in the puerperium because of a complication during birth, from 1st January 2015 to 31st December 2020.

### Data collection

Data were collected from admission registers of the department of obstetrics and gynaecology at the BRH, theatre registers and files from surgical wards, using a pre-designed data collection form. Collected data included, socio-demographic characteristics (age, antenatal visits, referral status), obstetric history (parity, previous uterine scar, curettage), type of delivery (vaginal or caesarean), clinical indicators (uterine rupture, intractable post-partum hemorrhage, uterine atony) and possible outcomes (sepsis, DIC, wound infection, acute kidney injury) of emergency obstetric hysterectomies.

### Statistical analysis

The collected data was entered into and analysed with EPI-INFO 7.2.2.1.

#### Dependent variable

Indications (intractable postpartum hysterectomy, uterine atony, abnormal placentation and uterine rupture) and outcomes (sepsis, anaemia, acute kidney injury and wound infection) of EOH were outlined and the frequency and percentages at which they occurred were calculated.

#### Independent variables

Age, number of antenatal visits, referral status, type of surgery, parity.

Predictors of adverse outcomes were identified using bivariate analysis, and the predictor variables were grouped into sociodemographic or obstetric. Chi-squared test was used to establish association between variables and to compare the two groups (CH and PH). A *P*-value < 0.05 was considered statistically significant.

### Ethics considerations

The ethical clearance for this study was issued by the institutional Review Board of the Faculty of Health Sciences, University of Buea (ref. N^o:^ 2019/1061–01/UB/SG/IRB/FHS). An administrative approval was obtained from the Directorate of the Bafoussam Regional Hospital, Cameroon (ref. N^o:^ 297/L/MINSANTE/SSG/DRSPO/HRB/D). To ensure confidentiality, all patient information was coded.

## Results

A total of 7992 medical records were reviewed and there were 6364 vaginal and 1634 caesarean deliveries. Of these, 32 medical records were obstetric hysterectomies. Two cases of elective hysterectomies because of symptomatic uterine fibroids and cervical cancer in pregnancy were excluded from the study. There was no exclusion of cases on the basis of incomplete or missing data, giving a retrieval rate of 100%. Therefore, 30 medical records of emergency obstetric hysterectomy (EOH) were included in the study; 24 caesarean hysterectomies (CH) and 6 postpartum hysterectomies (PH) (Table 1). Of the 30 cases, 10% (3/30) were referred from other hospitals for better management (Table 1).

**Table 1:** Types of emergency obstetric hysterectomy (EOH)

Type of EOH	Cases (%)	Referred Cases (%)	deliveries	Prevalence Per 1000
Caesarean hysterectomy	24 (80)	2 (6.67)	1628	14.7
Postpartum hysterectomy	6 (20)	1 (1.33)	6364	0.9
Total	30 (100)	3 (10)	7992	3.75

*EOH* Emergency obstetric hysterectomy

### Characteristics of the study population

The median age was 31.5 years (IQR: 27–36 years). Thirteen (43.33%) of cases were less than 30 years old. The median parity was 4 (IQR:2–5). Grand multiparity (*5 or more births*) accounted for 30% (9/30) of cases while multiparity *(2 to 4 births)* and primiparity accounted for 53.33% (16/30) and 16.67% (5/30) respectively. Out of 30 cases, 93.3% (28/30) attended ANC at least four times as against 6.7% (2/30) who did attend ANC less than four times (Table 2).

**Table 2:** Sociodemographic characteristics of EOH

Variable	Frequency	***Percentage (%)***
***Maternal age (years)***		
*< 20*	*2*	*6.7*
*20–30*	*13*	*43.3*
*> 30*	*15*	*50.0*
***Parity***		
*Primipara*	*5*	*16.7*
*2–4*	*16*	*53.3*
*≥ 5*	*9*	*30.0*
***ANC visits***		
*≥ 4*	*28*	*93.3*
*< 4*	*2*	*6.7*

*ANC* antenatal care

### Prevalence of emergency obstetric hysterectomy, postpartum hysterectomy and caesarean hysterectomy

The prevalence of emergency obstetric hysterectomy was 3.75 per 1000 livebirths (30/7992). Twenty-four cases underwent hysterectomy after caesarean deliveries 24/7992 (prevalence of 3.0/1000 livebirths) and six after vaginal deliveries 6/7992 (prevalence of 0.75/1000 livebirths).

### Characteristics of emergency obstetric hysterectomy

Multiparity occurred in 25 (83.33%) cases. Multiparity (25), uterine atony (10), were more likely to lead to caesarean hysterectomy (CH) than postpartum hysterectomy (PH) (Table 3).

**Table 3:** Characteristic of emergency obstetric hysterectomy cases (CH vs PH)

Risk factors	Frequency of EOH (%)	CH N (%)	PH N (%)
Multiparity	25 (83.33)	21 (87.50)	4 (66.67)
Uterine atony	10 (33.33)	9 (37.50)	1 (16.67)
Previous uterine scar	8 (26.67)	8 (33.33)	0 (00)
Previous uterine curettage	5 (16.67)	5 (12.50)	3 (33.33)
Placenta accreta	4 (13.33)	3 (12.50)	1 (16.67)
Coagulopathy	3 (10.00)	3 (12.50)	0 (00)
Uterine rupture	2 (6.67)	2 (8.33)	0 (00)

*CH* caesarean hysterectomy, *PH* postpartum hysterectomy, *EOH* emergency obstetric hysterectomy

### Indications of emergency obstetric hysterectomies

Intractable postpartum haemorrhage 10 (33.33) was the most frequent indication of EOH, with the same odds of being an indication for both postpartum hysterectomy and caesarean hysterectomy (Table 4).

**Table 4:** Indications of EOH

Indications	Frequency of EOH (%)	CH N (%)	PH N (%)
Intractable PPH	10 (33.33)	8 (33.3)	2 (33.3)
Uterine Atony	9 (30)	7 (29.17)	2 (33.33)
Abnormal Placentation	8 (26.67)	5 (20.83)	3 (50)
Uterine rupture	8 (26.67)	8 (33.3)	0 (00)

*CH* caesarean hysterectomy, *PH* postpartum hysterectomy, *EOH* emergency obstetric hysterectomy, *PPH* postpartum haemorrhage

### Complications of emergency obstetric hysterectomy

The most frequent complication of EOH was anaemia (27, 90%). Other than sepsis which had a significant difference in both caesarean hysterectomies and postpartum hysterectomies, there were no statistically significant differences for anaemia, AKI and wound infections. There was an overall 1 (3.85%) mortality, 1 (3.85%) for CH and 0 (00%) for PH, but there was no statistically significant difference in mortality rate between CH and PH (Table 5).

**Table 5:** Complications of EOH

Complications of EOH	Frequency of EOH (%)	CH N (%)	PH N (%)
Anaemia	27 (90)	21 (87.50)	6 (100)
Sepsis	2 (6.67)	0 (00)	2 (33.3)
AKI	1 (3.33)	1 (4.17)	0 (00)
Wound infection	2 (6.67)	2 (8.33)	0 (00)
Death	1 (3.33)	1 (3.33)	0 (00)

*EOH* emergency obstetric hysterectomy, *AKI* Acute kidney injury, *PH* Postpartum hysterectomy, *CH* Caesarean hysterectomy

### Predictors of adverse clinical outcomes of emergency obstetric hysterectomy

Age, parity, referral status, transfusion prior to surgery, type of surgery and method of delivery were evaluated as predictors of adverse clinical outcomes. In bivariate analysis, participants who did not receive blood transfusion prior to surgery were 25-fold more likely to be anaemic after EOH (OR 25; 95%CI: 1.52–410.89; *p* = 0.04) (Table 6).

**Table 6:** Predictors of adverse clinical outcome

Variables	Total n (%) ***N*** = 30	Adverse outcome n (%) ***n*** = 28	No adverse outcome n (%) ***n*** = 2	Risk ratio	Confidence interval	***p***-value
**Age (years)**						
< 30	13 (100)	11 (84.62)	2 (15.38)	1.18	0.94–1.49	0.18
≥ 30	17 (100)	17 (100)	0 (00)			
**Multiparity**						
Yes	25 (100)	24 (96)	1 (4.00)	1.2	0.77–1.87	0.31
No	5 (100)	4 (80)	1 (20.00)			
**ANC**						
Yes	27 (100)	25 (92.59)	2 (7.41)	0.9	0.83–1.03	1.00
No	2 (100)	2 (100)	0 (00)			
**Referred**						
Yes	3 (100)	3 (100)	0 (00)	1.1	0.97–1.2	1.00
No	27 (100)	25 (92.59)	2 (7.41)			
**Blood transfusion**						
Yes	4 (100)	2 (50)	2 (50)	0.5	0.19–1.33	0.013*
No	26 (100)	26 (100)	0 (00)			
**Type of surgery**						
Subtotal	27 (100)	25 (92.59)	2 (7.41)	0.9	0.83–1.03	1.00
Total	1 (100)	1 (100)	0 (00)			
**Method of delivery**						
C/S	24 (100)	22 (91.67)	2 (8.33)	0.9	0.81–1.03	1.00
VD	6 (100)	6 (100)	0 (00)			
**Duration of surgery (hours)**						
< 2	6 (100)	4 (66.67)	2 (33.33)	0.67	0.38–1.17	0.04*
≥ 2	22 (100)	22 (100)	0 (00)			

*ANC* antenatal care, *CS* Caesarean section, *VD* vaginal delivery

* Statistically significant values

## Discussion

We observed an overall high prevalence rate of EOH (1 in about 267 deliveries). This is higher than the 1.25 per 1000 deliveries and the 1.14 per 1000 deliveries reported in two tertiary hospitals in Yaounde and Douala, Cameroon [6, 12]. This difference could be due to the fact that both Douala and Yaounde teaching hospitals are tertiary hospitals and more equipped to monitor and handle deliveries hence preventing complications which could require EOH as last resort. Furthermore, the Douala and Yaounde teaching hospitals have more experienced medical personnel to better perform deliveries. Our prevalence of 1 in 267 deliveries is however within the global range [13] and similar to the 5.1 per 1000 deliveries reported by Nwobodo et al. [5] at a tertiary hospital in Sokoto, Nigeria. The prevalence was higher than the 1 in 1429 deliveries reported in upper middle and high income countries [4]. This could be due to better obstetric care in such settings. Among the 30 cases of EOH, more than 3 in 4 were caesarean hysterectomy which was similar to reports (70) by Forna et al. [14]. Multiparity (83.33%) was the most frequent risk factor in our study. This was similar to findings from a system review by Rossi et al in 2010 [15]. Also, reports by Njamen et al in 2017, indicated that, every 3 in 4 cases of EOH, involved a multiparous woman [12]. Multiparous patients were more than 3 times more like to have a CH than PH. There was no statistically significant difference, drawing similarity with a study done by Lee and To in Hong Kong [16]. Intractable postpartum haemorrhage (33.3%) followed by Uterine atony (30%) were the most common indication of EOH, this finding ties with that in studies by Forna et al. [14] in the US and Njamen et al. [12] in Cameroon. Comparison of indications of emergency obstetric hysterectomy revealed that the primary cause of CH was intractable postpartum haemorrhage (33.33%) and uterine rupture (33.33%) and in the PH group, abnormal placentation (50%) was the primary indication, similarly, to Forna et al. [14], but there were no significant differences in the indications of CH and PH. Approximately 9 in every 10 patients had a complication following emergency obstetric hysterectomy. The most frequent complication of EOH was anaemia which occurred 9 times in every 10 patients. This could be due to the delay in acquiring blood as in our setting, the blood banks are frequently dried out, and getting a donor is daunting task, ranging from identification to pre-transfusion laboratory workups which take a lot of time. Emergency obstetric hysterectomy patients who delivered vaginally had a statistically higher chance of developing sepsis when compared to those who underwent EOH following caesarean section. This could be attributed to the fact that the delivery rooms in our settings are relatively more septic than the theatres as there is little knowledge and application of basic hygiene measures in our labour rooms by nurses as compared to doctors in the theatre. Furthermore, vaginal deliveries entail a longer duration and require multiple vaginal examinations which is significantly associated with intraamniotic infections which could lead to sepsis [17, 18]. Preoperative antibiotics prophylaxis is significantly associated with reduced wound infection which is a cause of sepsis [19–21], but in our setting, there is usually no administration of prophylactic antibiotic therapy during vaginal delivery as recommended in caesarean section. Again, EOH following vaginal delivery is usually followed by multiple desperate emergency maneuvers like uterine curettage, uterine packing and insertion of utero-tonics in attempt to save the woman’s life which increases the chances of sepsis. Recognition of pre-operative predictors of adverse outcomes such as: age, parity, referral status, duration of surgery, type of surgery, duration from indication to surgery, blood transfusion, method of delivery were assessed. Patients who weren’t transfused prior to surgery had a significant chance of having an adverse outcome. Patients who were not transfused were 25 times more likely to have anaemia after surgery. Also, patients whose surgery lasted 2 h and more were significantly more likely to develop an adverse outcome. A possible reason for this, could be prolonged bleeding, hence anaemia. In our study, 9 in every 10 patients had anaemia. Extensive probe of existing literature for comparative analysis, to the best of our knowledge proved abortive, with respect to finding studies that assessed predictors of adverse outcomes of EOH.

### Strengths of the study

To the best of our knowledge, this is one of the few studies in Africa that compares and brings out the difference and similarities in the clinical profile, indications and outcomes of caesarean hysterectomy (CH) and postpartum hysterectomy (PH). Additionally, our study contributes to the data on the prevalence, indications and outcomes of emergency obstetric hysterectomies in Cameroon and the world at large. Furthermore, our study provides new data on clinical outcomes and the predictors of these outcomes.

### Limitations of the study

The sample size for this study was small thereby warranting studies with larger sample sizes. Furthermore, due to the retrospective design of the study, there was a possibility of getting inadequate or missing data in the medical records of some patients.

## Conclusion

About 1 in 267 women who gave birth at the Bafoussam regional hospital underwent an emergency obstetric hysterectomy. Multiparity, intractable postpartum haemorrhage, and anaemia were the significant risk factor, indication and complication, respectively. Sepsis was significantly different between caesarean hysterectomy and postpartum hysterectomy. Cases that did not receive blood transfusion prior to surgery were more likely to present with anaemia. Despite being a life-saving endeavour, rendering a young woman sterile could have profound psycho-social effects. Efforts towards preventing identified risk factors and indications thus becomes vital in order to curb high rates of EOH and sterility which induces a high psycho-social burden on our young women.

## Data Availability

The data sets supporting the findings of this study are available, and can be provided by the corresponding author on reasonable request.

## Abbreviations

ARDS: Acute respiratory distress syndrome
ANC: Antenatal clinic
BRH: Bafoussam Regional Hospital
CS: Caesarean section
CH: Caesarean hysterectomy
DIC: Disseminated intravascular coagulation
EOH: Emergency obstetric hysterectomy
EPH: Emergency peripartum hysterectomy
ICU: Intensive care unit
IQR: Interquartile range
PH: Postpartum hysterectomy
PPH: Post-partum haemorrhage
VD: Vaginal delivery
WHO: World Health Organization
